# Biosynthesis and biological function of secondary metabolites of the rice blast fungus *Pyricularia oryzae*

**DOI:** 10.1093/jimb/kuab058

**Published:** 2021-08-11

**Authors:** Takayuki Motoyama, Choong-Soo Yun, Hiroyuki Osada

**Affiliations:** Chemical Biology Research Group, RIKEN Center for Sustainable Resource Science, Wako, Saitama 351-0198, Japan; Chemical Biology Research Group, RIKEN Center for Sustainable Resource Science, Wako, Saitama 351-0198, Japan; Chemical Biology Research Group, RIKEN Center for Sustainable Resource Science, Wako, Saitama 351-0198, Japan

**Keywords:** Rice blast fungus *Magnaporthe oryzae*, Plant pathogenic fungus, Secondary metabolite biosynthetic gene cluster, Biological function

## Abstract

Filamentous fungi have many secondary metabolism genes and produce a wide variety of secondary metabolites with complex and unique structures. However, the role of most secondary metabolites remains unclear. Moreover, most fungal secondary metabolism genes are silent or poorly expressed under laboratory conditions and are difficult to utilize. *Pyricularia oryzae*, the causal pathogen of rice blast disease, is a well-characterized plant pathogenic fungus. *P. oryzae* also has a large number of secondary metabolism genes and appears to be a suitable organism for analyzing secondary metabolites. However, in case of this fungus, biosynthetic genes for only four groups of secondary metabolites have been well characterized. Among two of the four groups of secondary metabolites, biosynthetic genes were identified by activating secondary metabolism. These secondary metabolites include melanin, a polyketide compound required for rice infection; tenuazonic acid, a well-known mycotoxin produced by various plant pathogenic fungi and biosynthesized by a unique nonribosomal peptide synthetase–polyketide synthase hybrid enzyme; nectriapyrones, antibacterial polyketide compounds produced mainly by symbiotic fungi, including plant pathogens and endophytes, and pyriculols, phytotoxic polyketide compounds. This review mainly focuses on the biosynthesis and biological functions of the four groups of *P. oryzae* secondary metabolites.

## Introduction

Filamentous fungi, including plant pathogenic fungi, produce a wide variety of secondary metabolites with complex and unique structures. However, the biological role of secondary metabolites remains unclear. Filamentous fungi have been a rich source of secondary metabolites for medicines and agrochemicals. Whole-genome sequencing analyses have indicated that filamentous fungi have many more secondary metabolism genes than expected, suggesting that most secondary metabolism genes are silent or poorly expressed under laboratory conditions. To utilize the ability of fungi to produce secondary metabolites, secondary metabolism has been activated through various approaches, including overexpression of pathway-specific transcription factors, manipulation of global regulators, epigenetic control, ribosome engineering, heterologous expression of secondary metabolite biosynthetic gene clusters (BGCs), coculture, and chemical induction (Macheleidt et al., 2016; Netzker et al., 2015; Ochi & Hosaka, 2013; Rutledge & Challis, 2015). A recent study showed that rice extracts can induce secondary metabolite gene expression in the filamentous fungus *Aspergillus nidulans* (Lacriola et al., 2020), suggesting the possible involvement of fungal secondary metabolites in plant–fungus interactions.

The major fungal secondary metabolites, polyketides and nonribosomal peptides, are biosynthesized by polyketide synthases (PKSs) and nonribosomal peptide synthetases (NRPSs), respectively. Fungal PKSs can be categorized into three types. The first type is iterative type I PKS that consists of multiple catalytic domains, including ketosynthase (KS), acyltransferase (AT), and acyl carrier protein (ACP) domains, along with several optional β-keto modifying domains, such as β-ketoacyl reductase (KR), dehydratase (DH), and transacting enoyl reductase domains (Fischbach & Walsh, 2006). The second type is type III PKS, which consists of only homodimeric KS (Hashimoto et al., 2014). The third type is a fungal PKS–NRPS hybrid enzyme, which consists of an iterative type I PKS followed by a single-module NRPS (Boettger & Hertweck, 2013; Böhnert et al., 2004; Eley et al., 2007; Fisch, 2013; Song et al., 2004). In fungal PKS–NRPS, the PKS part has KS, AT, and ACP domains, along with several modifying domains including KR, DH, and methyltransferase (MT) domains. The NRPS part has adenylation (A), peptidyl carrier protein (PCP), condensation (C), and terminal release or cyclization (R, reductase or DKC, Dieckmann cyclization) domains (Boettger & Hertweck, 2013).

**Fig. 1.**
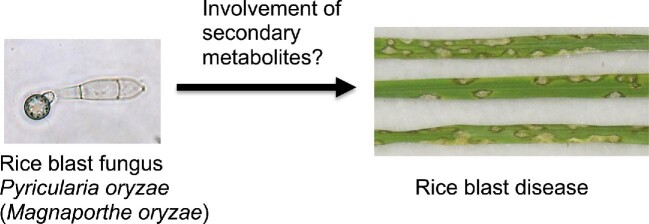
Are secondary metabolites of the rice blast fungus *Pyricularia oryzae* involved in rice infection?

**Fig. 2.**
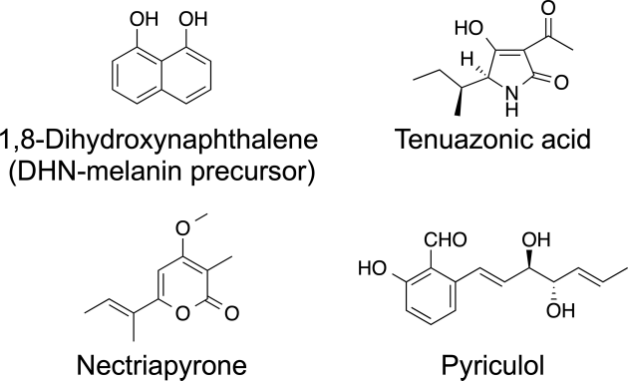
Chemical structures of the secondary metabolites of *Pyricularia oryzae*.

*Pyricularia oryzae* (syn. *Magnaporthe oryzae*) (Fig. 1) is the causal pathogen of rice blast disease and is a well-characterized plant pathogen. *P. oryzae* infects rice plants through an infection-specific organ appressorium and proliferates inside rice plants via filamentous growth and causes rice blast disease (Howard & Valent, 1996). *P. oryzae* is also rich in secondary metabolism genes (Collemare et al., 2008b; Dean et al., 2005). An antiSMASH analysis predicted the presence of 23 iterative type I PKSs, 2 type III PKSs, 6 fungal PKS–NRPS hybrid enzymes, and 9 NRPSs (unpublished data). Secondary metabolites of *P. oryzae* may be involved in rice infection (Fig. 1). The biosynthetic genes of only four groups of secondary metabolites (melanin, tenuazonic acid, nectriapyrones, and pyriculols) have been well characterized in *P. oryzae* (Fig. 2). Two (tenuazonic acid and nectryapyrones) of the four groups of secondary metabolites were identified by activating secondary metabolism.

Here, we mainly focused on the four groups of secondary metabolites, as shown in Fig. 2. We describe the biosynthesis and biological roles of secondary metabolites in the rice blast fungus *P. oryzae*.

## Melanin: A Polyketide Compound Required for the Fungal Infection to Rice

*P. oryzae* synthesizes the black pigment melanin (Fig. 2), which is required for rice infection (Howard & Ferrari, 1989). Melanin does not work as a toxin, but this secondary metabolite is required for rice infection by the mechanism described below. *P. oryzae* forms an infection-specific organ, appressorium, and infects rice plants using this organ. Appressorium formation and appressorium melanization are essential for rice infection. Rice plant invasion is achieved by an infection peg that is formed at the base of an appressorium, which attaches tightly to the host surface. The mechanical force produced by appressoria is necessary to successfully penetrate the infection peg (Howard & Ferrari, 1989). An appressorial melanin layer between the cell membrane and the cell wall is essential for mechanical force generation. Turgor pressure is concentrated toward the cuticular surfaces of the rice plant, and the pressure inside the appressoria has been assessed to be as high as 8 MPa (Howard et al., 1991; Money & Howard, 1996). This pressure can be generated by 3.2 M glycerol accumulated inside the appressorium (de Jong et al., 1997). Melanin is proposed to function as a semipermeable membrane that passes water, but not glycerol, and as a structural support for this extremely high pressure.

**Fig. 3.**
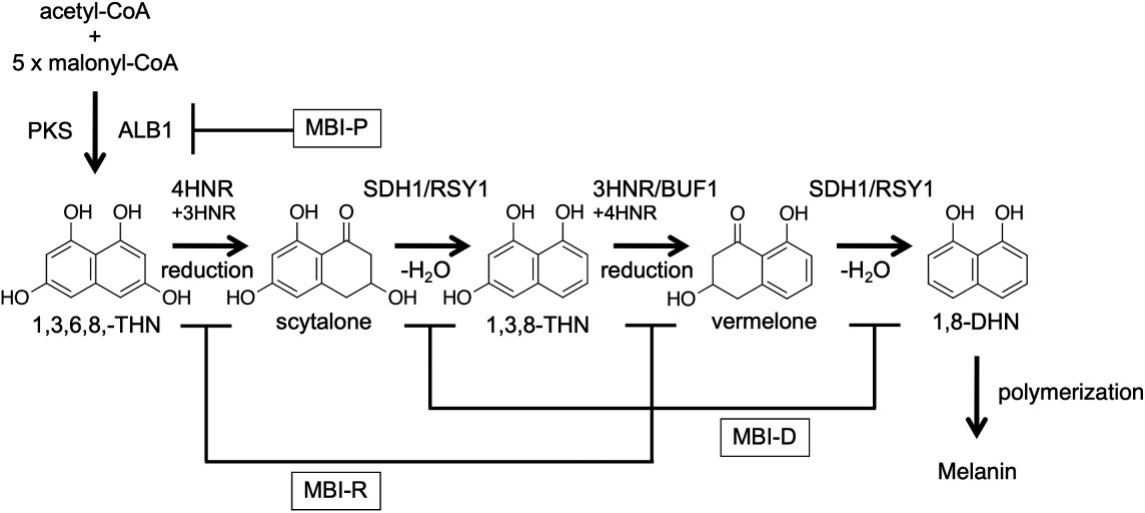
Melanin biosynthetic pathway of *Pyricularia oryzae*. Inhibition points of the three groups of melanin biosynthesis inhibitors (MBIs) are shown. 1,3,6,8-THN, 1,3,6,8-tetrahydroxynaphthalene; 1,3,8-THN, 1,3,8-trihydroxynaphthalene; 1,8-DHN, 1,8-dihydroxynaphthalene.

Melanin is a well-known natural black pigment. *P. oryzae* produces dihydroxynaphthalene (DHN)-melanin, which is a type of fungal melanin biosynthesized by polymerizing the polyketide compound 1,8-dihydroxynaphthalene (1,8-DHN) (Bell & Wheeler, 1986; Butler & Day, 1998). In *P. oryzae*, melanin biosynthetic genes have been identified, and their biosynthetic pathways have been elucidated (Fig. 3) (Eliahu et al., 2007; Lundqvist et al., 1994; Thompson et al., 2000; Vidal-Cros et al., 1994). The iterative type I PKS enzyme ALB1/MGG_07219 catalyzes the synthesis of the backbone compound 1,3,6,8-tetrahydroxynaphthalene (1,3,6,8-THN). Melanin was originally shown to be a pentaketide compound. However, analysis of an ALB1 homolog in the closely related fungus *Colletotrichum lagenarium* showed that melanin is a hexaketide compound and the backbone (1,3,6,8-THN) is biosynthesized using acetyl-CoA and five malonyl-CoA (Vagstad et al., 2012). Then, 1,3,6,8-THN is converted to 1,8-DHN using three enzymes: 1,3,6,8-THN reductase (4HNR), scytalone dehydratase (SDH1/RSY1), and 1,3,8-trihydroxynaphthalene (1,3,8-THN) reductase (3HNR/BUF1). Finally, 1,8-DHN is oxidatively polymerized to form DHN-melanin. Melanin biosynthesis can be induced by genetic modification of the factors involved in epigenetic control (Maeda et al., 2017) and signal transduction (Motoyama et al., 2008).

Biosynthetic enzymes of DHN-melanin have been agrochemical development targets, and three types of commercial melanin biosynthesis inhibitors (MBIs) have been successfully developed (Fig. 3). MBIs are classified into three types: MBI-R (tricyclazole, phthalide, and pyroquilon), MBI-D (carpropamid, fenoxanil, and diclocymet), and MBI-P (tolprocarb) (Banba et al., 2017; Hagiwara et al., 2019; Motoyama & Yamaguchi, 2003). The targets of MBI-R, MBI-D, and MBI-P are 1,3,8-THN reductase (3HNR/BUF1), scytalone dehydratase (SDH1/RSY1), and PKS (ALB1), respectively (Fig. 3). MBIs are known to be environmentally friendly agrochemicals because they can inhibit fungal infection without inhibiting fungal growth. As MBIs have no growth inhibitory activity, it has long been believed that no resistant strain will be created. However, the occurrence of resistant strains for MBI-D has been reported (Kaku et al., 2003; Sawada et al., 2004; Yamada et al., 2004), suggesting that resistant strains can become dominant depending on the target enzymes and application conditions.

## Tenuazonic Acid: A Well-Known Mycotoxin Biosynthesized by a Unique NRPS–PKS Enzyme

The tetramic acid derivative tenuazonic acid (TeA, Fig. 2) is a well-known mycotoxin that was first isolated from *Alternaria tenuis* culture broth in 1957 (Rosett et al., 1957). *P. oryzae* and other plant pathogenic fungus *Phoma sorghina* (sorghum pathogen) are also known as TeA producers (Steyn & Rabie, 1976; Umetsu et al., 1972). *Alternaria* is a ubiquitous plant pathogenic fungus that causes spoilage of various food crops and fruits in the field and postharvest decay (Ostry, 2008). TeA has been detected in many *Alternaria*-contaminated vegetables, fruits, and crops (Gross et al., 2011; Lohrey et al., 2013; Siegel et al., 2009). A TeA analog from valine is also produced by *P. oryzae* as a minor product (Lebrun et al., 1990). Among the *Alternaria* toxins, TeA showed the highest toxicity, with acute toxicity in mammals. The oral median lethal doses for male and female mice were 182 or 225 mg kg^−1^ and 81 mg kg^−1^ body weight, respectively (Miller et al., 1963; Smith et al., 1968). The European Food Safety Authority determined the threshold of toxicological concern of TeA to be 1500 ng kg^−1^ body weight (Asam & Rychlik, 2013).

TeA inhibits protein synthesis by preventing the release of polypeptides from the ribosome (Shigeura & Gordon, 1963). TeA also shows antiviral, antibacterial, antitumor, phytotoxic, and plant disease-controlling activities (Aver'yanov et al., 2007; Gitterman, 1965; Lebrun et al., 1988; Miller et al., 1963). TeA can inhibit photosynthesis (Chen et al., 2015, 2007, 2010) and the potential use of TeA as a herbicide that targets photosystem II (PSII) has been proposed (Chen & Qiang, 2017). A recent study indicated that TeA can inhibit plant plasma membrane (PM) H^+^-ATPase at micromolar concentrations (Bjørk et al., 2020). PM H^+^-ATPase inhibition results in depolarization of the membrane potential and finally necrosis. Currently, it remains unknown whether TeA is required for plant infection. We induced the production of TeA in *P. oryzae* and identified the biosynthetic gene *TAS1*, which encodes the first reported fungal NRPS–PKS hybrid enzyme (Motoyama & Osada, 2016; Yun et al., 2015). We have shown the biosynthetic and induction mechanisms of TeA (Yun et al., 2017, 2020). In the following section, we present the data on the biosynthesis and induction of TeA.

**Fig. 4.**
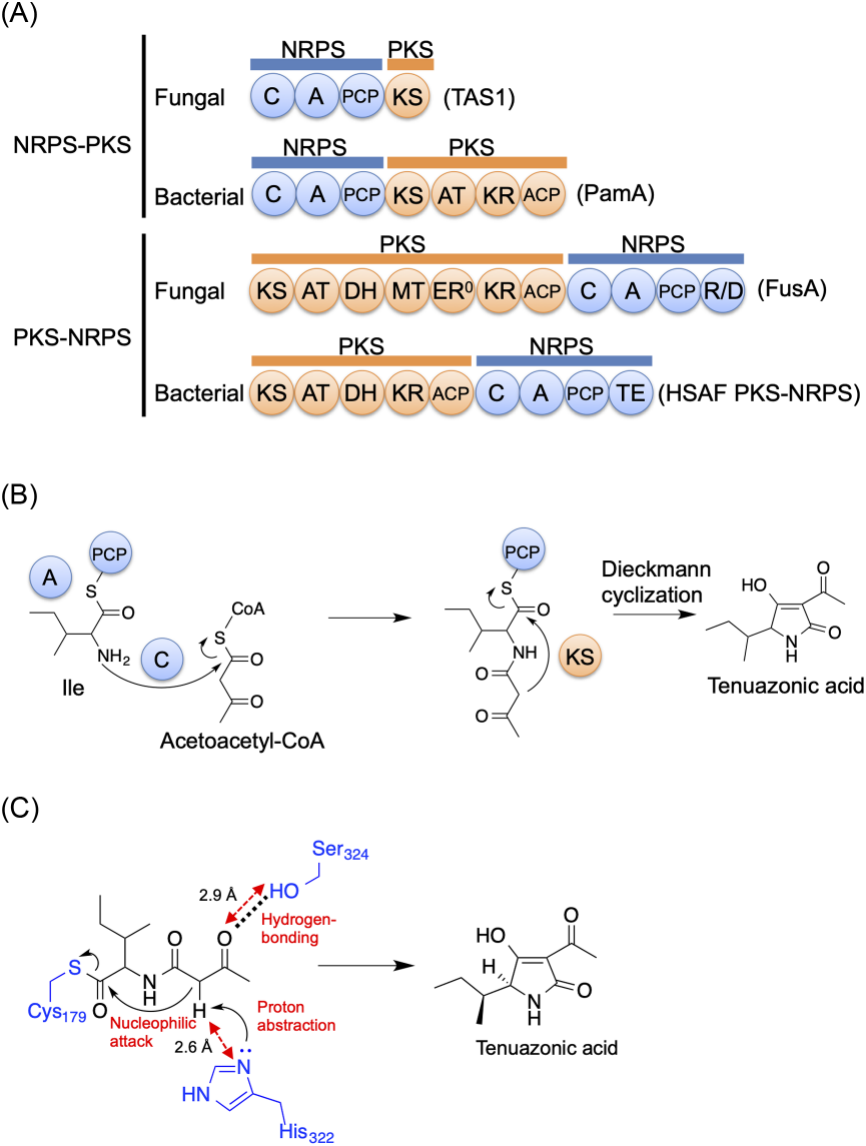
Tenuazonic acid biosynthesis. (A) Comparison of the domain structures of TAS1 and other enzymes. (B) Biosynthetic pathway of tenuazonic acid. (C) Proposed cyclization mechanism for tenuazonic acid. KS, ketosynthase; NRPS, nonribosomal peptide synthetase; PKS, polyketide synthase; TAS1, tenuazonic acid synthetase 1.

TeA has been shown to be biosynthesized using an isoleucine and two acetates (Stickings & Townsend, 1961). TeA was also predicted to be a product of a PKS–NRPS hybrid enzyme because it has a tetramic acid-containing structure (Collemare et al., 2008a). We successfully induced TeA production by 1% dimethyl sulfoxide (DMSO) treatment and disruption of the *OSM1* gene, which encodes an osmosensory mitogen-activated protein kinase (MAPK) that works downstream of the two-component signal transduction system involved in environmental responses. We found that the TeA biosynthetic gene *TAS1/MGG_07803* was upregulated under TeA-inducing conditions (Motoyama & Osada, 2016; Yun et al., 2015). Unexpectedly, the biosynthetic enzyme TAS1 (tenuazonic acid synthetase 1) was not a PKS–NRPS hybrid enzyme but an NRPS–PKS hybrid enzyme. PKS–NRPS hybrid enzymes have also been found in bacteria (Blodgett et al., 2010; Yu et al., 2007). Moreover, a different type of hybrid enzymes, NRPS–PKS hybrid enzymes (which begin with an NRPS module), have been found in bacteria (Du et al., 2001; Gerc et al., 2012; Müller et al., 2014; Silakowski et al., 1999; Simunovic et al., 2006; Tang et al., 2004). We reported that TAS1 is the first fungal NRPS–PKS hybrid enzyme, which consists of an NRPS module of domains C-A-PCP and a terminal PKS KS domain (Fig. 4A). TAS1 is a new type of NRPS–PKS hybrid enzyme that starts with an NRPS module (C-A-PCP). The domain structure is quite different from that of conventional fungal PKS–NRPS enzymes that start with a PKS module (Fig. 4A). The PKS part of TAS1 only has a KS domain, in contrast to other NRPS–PKS hybrid enzymes. This KS domain has a unique sequence and is essential for TAS1 activity. Phylogenetic analysis revealed that this KS domain formed an independent clade close to the type I PKS KS clade. We elucidated that TAS1 biosynthesizes TeA from isoleucine and acetoacetyl-CoA (diketide) (Fig. 4B). This unique KS domain catalyzes the final Dieckmann cyclization step for tetramic acid ring formation and TeA release, although previous studies have suggested the involvement of this KS domain in diketide biosynthesis (Stickings & Townsend, 1961). In contrast, fungal NRPSs use the terminal condensation-like domain for substrate cyclization (Gao et al., 2012) and bacterial NRPSs use the terminal thioesterase domain for substrate cyclization (Trauger et al., 2000). Moreover, fungal PKS–NRPSs use the terminal reductase-like cyclization (DKC) domain for substrate cyclization (Boettger & Hertweck, 2013). This information shows that TAS1 is a unique type of biosynthetic enzyme that may be used for the production of various compounds.

The PKS KS domains usually conduct the decarboxylative Claisen condensation of acyl and malonyl blocks to extend the polyketide chain (Hertweck, 2009). In contrast, the terminal KS domain of *P. oryzae* TAS1 catalyzes substrate cyclization (Yun et al., 2015). Interestingly, a KS domain with a cyclization and not ketide extension role has only been reported in TAS1. We analyzed the unique features of the KS domain of TAS1 (Yun et al., 2020). We revealed that the KS domain of TAS1 is unexpectedly monomeric such as NRPSs (Tanovic et al., 2008) although KSs are normally dimeric (Dutta et al., 2014; Kao et al., 1996; Weissman, 2015). The 1.68 Å resolution crystal structure of the TAS1 KS domain suggested that substrate cyclization is initiated by proton abstraction from the active methylene moiety in the substrate by the catalytic residue H322 (Fig. 4C). We also revealed that TAS1 KS shows broad substrate specificity and promiscuously accepts aminoacyl substrates. Moreover, this behavior could be increased by a single amino acid change in the substrate-binding pocket. Our data provided insight into the substrate cyclization mechanism catalyzed by the KS domain in TeA biosynthesis and how the KS domain in the NRPS–PKS hybrid enzyme TAS1 receives large amino acid-containing substrates.

**Fig. 5.**
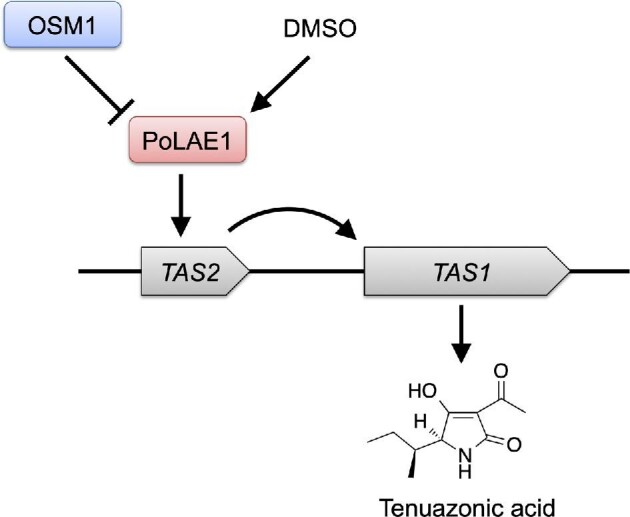
Regulation of tenuazonic acid production in *Pyricularia oryzae*.

The expression of fungal secondary metabolism genes should be properly regulated in response to various environmental signals. To understand the interaction between fungi and their environments, it is important to elucidate the regulatory mechanisms of these secondary metabolism genes. For example, clarification of the regulatory mechanism of mycotoxin biosynthesis is important to protect human and animal health by inhibiting mycotoxin production. As explained previously, TeA is biosynthesized by TAS1 in *P. oryzae*, and its production is induced by 1% DMSO treatment or osmosensory MAPK gene (*OSM1*) deletion. However, the detailed regulatory mechanism of TeA production remains unknown. We identified two positive regulators of TeA production (Fig. 5) (Yun et al., 2017). Most fungal secondary metabolites are produced using BGCs. Some gene clusters have a cluster-specific DNA-binding binuclear Zn(II)_2_Cys_6_-type transcription factor, which is unique to fungi and stimulates the transcription of the clustered genes to generate a secondary metabolite (Keller et al., 2005). Transcription factor genes include *Fusarium sporotrichioides tri6* for trichothecene biosynthesis (Proctor et al., 1995), *A. nidulans aflR* for aflatoxin biosynthesis (Yu et al., 1996), *Aspergillus fumigatus gliZ* for gliotoxin biosynthesis (Bok et al., 2006), and *Monascus purpureus ctnA* for citrinin biosynthesis (Shimizu et al., 2007). We identified TAS2 (MGG_07800), a Zn(II)_2_Cys_6_-type transcription factor that regulates the production of TeA. *TAS2* is located in the upstream region of *TAS1* (Fig. 5). In fungi, the secondary metabolite production is regulated by upper-level regulators. These regulators are called global regulators, which are transacting positive or negative transcription factors of secondary metabolite BGCs. Loss of *aflR* expression (LaeA) is a well-characterized global regulator of secondary metabolism that was originally identified in *Aspergillus* spp. (Bayram et al., 2008; Brakhage, 2013; Perrin et al., 2007; Yin & Keller, 2011). LaeA orthologs have been identified in other fungi, including *Fusarium* spp., *Cochliobolus heterostrophus*, and *Monascus pilosus* (Bok & Keller, 2016). We identified the LaeA ortholog PoLAE1 (MGG_01233) from *P. oryzae*. Analysis of *PoLAE1* knockout and overexpression strains indicated that PoLAE1 positively controlled the production of TeA. We also found that the two TeA-inducing signals, 1% DMSO treatment and *OSM1* deletion, were transmitted through PoLAE1. These results showed that TeA production was regulated by two specific transcriptional regulators, TAS2 and PoLAE1, in *P. oryzae* (Fig. 5). Recently, it has been shown that TeA production can be induced by mycoviruses via upregulation of *TAS2* (Ninomiya et al., 2020).

## Nectriapyrones: Antibacterial Polyketide Compounds

Nectriapyrone (Fig. 2) is a polyketide compound, which is produced by various fungi (Andolfi et al., 2015; Avent et al., 1992; Claydon et al., 1985; Evidente et al., 2011; Gong et al., 2015; Guimaraes et al., 2008; He et al., 2016; Meister et al., 2007; Nair & Carey, 1975; Thines et al., 1998; Weber et al., 2005; Xi et al., 2012; Zhu et al., 2013). Interestingly, nectriapyrone producers are mainly symbiotic fungi, including plant pathogens (Andolfi et al., 2015; Evidente et al., 2011), endophytes (Claydon et al., 1985; Guimaraes et al., 2008; Lu et al., 2009; Meister et al., 2007; Weber et al., 2005; Xi et al., 2012), and sponge-associated fungi (Gong et al., 2015; He et al., 2016; Zhu et al., 2013). We recently revealed that nectriapyrone production can be induced in *P. oryzae* by disturbing the two-component signal transduction system (Motoyama et al., 2019). We succeeded in identifying the nectriapyrone BGC and analyzed its physiological functions, as described below.

The production of secondary metabolites is predicted to be strictly regulated under specific environmental conditions. Thus, we expected that secondary metabolite production might be activated by manipulating the signal transduction pathways involved in environmental responses. The two-component system (TCS) is a signal transduction system that controls various cellular functions in response to environmental signals and is found in various organisms, including bacteria, archaea, plants, slime molds, and fungi (Catlett et al., 2003; West & Stock, 2001). The *P. oryzae* TCS was disturbed by deletion *of OSM1* and *PoYPD1*, which encode HOG MAP kinase and His-containing phosphotransfer protein, respectively. This genetic manipulation induced the production of two polyketide compounds, nectriapyrone and its oxidized analog.

**Fig. 6.**
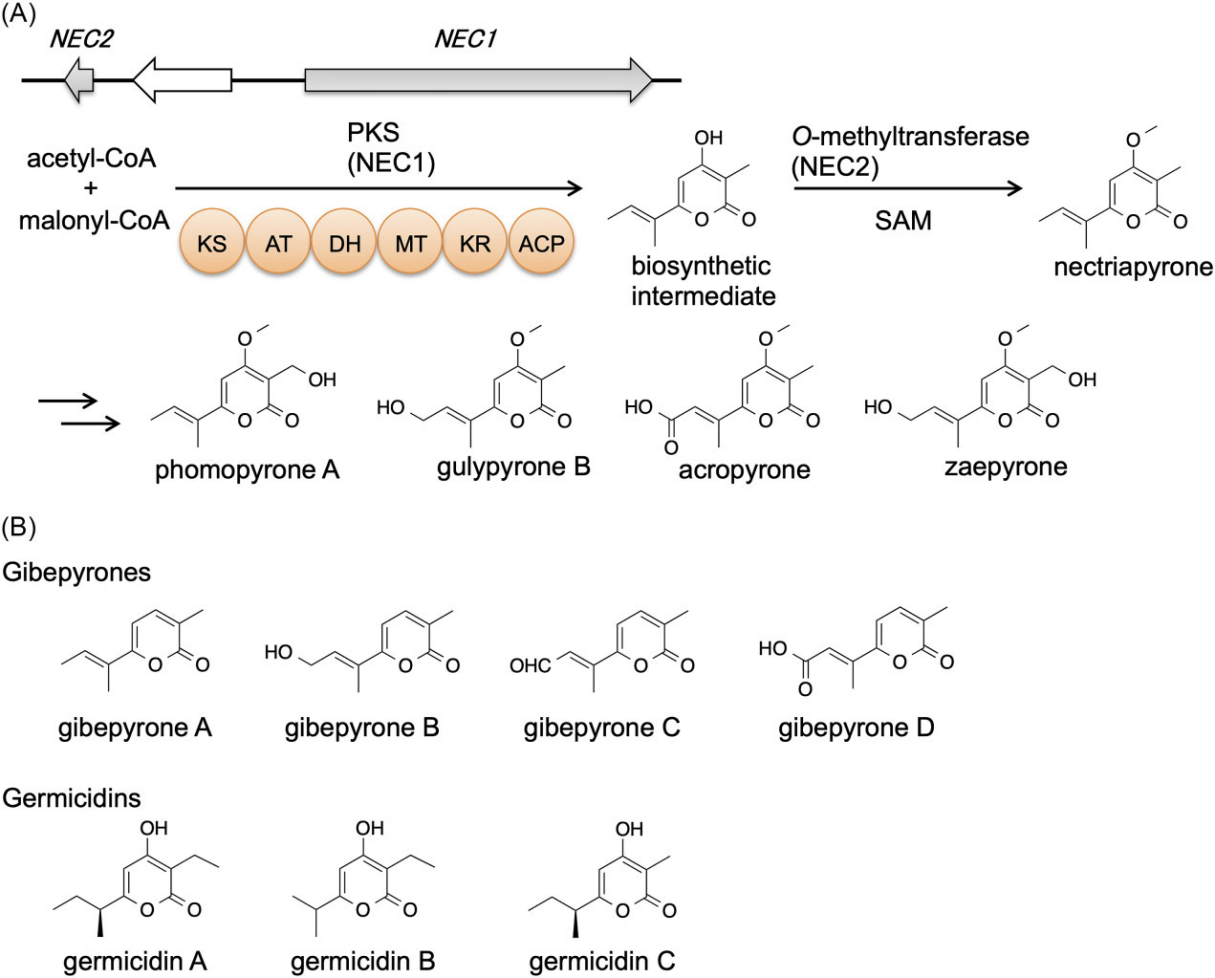
Nectriapyrones and related compounds. (A) Biosynthesis of nectriapyrones. (B) Related compounds of nectriapyrones.

We found a nectriapyrone BGC containing an iterative type I PKS gene (*NEC1*/*MGG_00806*) and an *O*-MT gene (*NEC2*/*MGG_14657*) (Fig. 6A). Overexpression of these two genes induced overproduction of nectriapyrone and five analogs, including a new derivative, zaepyrone (Fig. 6A) (Abramson & Wormser, 1981; Burkhardt & Dickschat, 2018; Cai et al., 2017; Hammerschmidt et al., 2014; Motoyama et al., 2019). Nectriapyrone is similar to gibepyrones (Fig. 6B) from *Fusarium* spp. Gebepyrones lack a methoxy group found in nectriapyrone, and the *O*-MT gene is absent from the gibepyrone BGC (Janevska et al., 2016). Nectriapyrone also shows similarity to the *Streptomyces* metabolite germicidins (Aoki et al., 2011; Petersen et al., 1993; Xu et al., 2011) (Fig. 6B). A type III PKS is known to biosynthesize the germicidin backbone (Song et al., 2006). In contrast, in case of nectriapyrone, a type I PKS (NEC1) biosynthesizes the nectriapyrone backbone.

Nectriapyrones are α-pyrone compounds that have a wide range of biological activities (McGlacken & Fairlamb, 2005; Schaberle, 2016). For example, germicidins, produced by *Streptomyces* strains, act as autoregulators of spore germination (Aoki et al., 2011; Petersen et al., 1993). Photopyrones are bacterial signaling molecules that control cell clumping (Brachmann et al., 2013). The biological activities of nectriapyrone have been reported, although the functions of nectriapyrone in its producers are unknown. Nectriapyrone is toxic to bacteria, plants, and tumor cells (Evidente et al., 2011; Guimaraes et al., 2008; Nair & Carey, 1975; Turkkan et al., 2011). Nectriapyrone stimulates the formation of DOPA (3,4-dihydroxyphenylalanine) melanin in B16-F1 melanoma cells (Thines et al., 1998). In addition, it has been reported to inhibit monoamine oxidase in the mouse brain (Lee et al., 1999).

Identification of the nectriapyrone BGC enabled us to analyze the biological roles of nectriapyrones in the fungus that produce them (Motoyama et al., 2019). Although many nectriapyrone producers have been identified from plant pathogens, we have revealed that nectriapyrones are not required for rice infection and have different roles. The chemical structure of nectriapyrone is similar to that of germicidins produced by *Streptomyces* spp. Our data showed that nectriapyrones can control growth and pigment formation in *Streptomyces griseus* and have a growth-promoting effect on *P. oryzae* in interactions with *S. griseus*. Therefore, nectriapyrones may be involved in microbe–microbe interactions with other environmental bacteria, such as endophytic *Streptomyces* strains. To identify the active nectriapyrone analogs in this interaction, we tested the bioactivity of each nectriapyrone analog (Fig. 6A) and showed that nectriapyrone was the only active analog, suggesting that other nectriapyrone analogs may be detoxified compounds of nectriapyrone.

## Pyriculols: Phytotoxic Polyketide Compounds

**Fig. 7.**
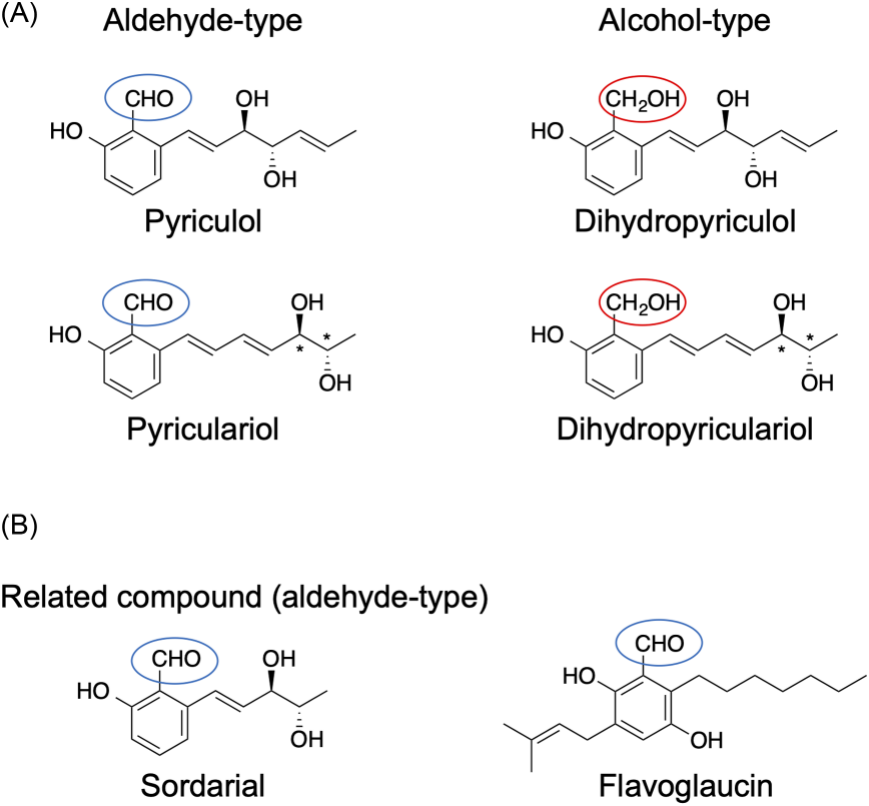
Structures of the pyriculols (A) and a related compound (B).

Pyriculol (Fig. 2) is a major secondary metabolite of *P. oryzae* and is a phytotoxin (Iwasaki et al., 1973). Several pyriculol analogs have also been reported. The major analogs are pyriculariol (Nukina et al., 1981), dihydropyriculol (Kono et al., 1991), and dihydropyriculariol (Tanaka et al., 2011b) (Fig. 7A). It is also reported that other pyriculol analogs, griseaketides, are produced by a rice blast fungus isolate (Yang et al., 2019). Pyriculols are classified into two groups: aldehyde-type (pyriculol and pyriculariol) and alcohol-type (dihydropyriculol and dihydropyriculariol). It has been shown that aldehyde-type analogs induce lesion-like necrosis in rice leaves, whereas alcohol-type analogs are inactive (Jacob et al., 2017; Kono et al., 1991; Tanaka et al., 2011b). Four analogs are produced simultaneously (Jacob et al., 2017), and interconversion between oxidized aldehyde-type analogs and reduced alcohol-type analogs is expected. It is important to know why and how *P. oryzae* produces both alcohol and aldehyde analogs. Identification of the genes responsible for this oxidoreductive conversion will help to answer this question. Here, we describe the identification of the pyriculol BGC and analyze the pyriculol function using the biosynthetic genes.

**Fig. 8.**
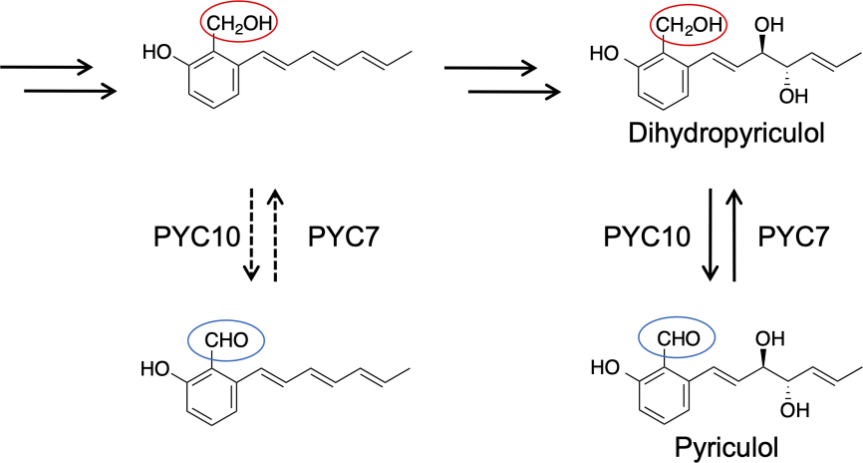
Interconversion of the two types of pyriculols.

Pyriculols are polyketide compounds, and their BGC have been reported (Furuyama et al., 2021a; Jacob et al., 2017). This gene cluster has an iterative type I PKS gene (*MGG_10912*/*MoPKS19*/*PYC1*) and other genes predicted to be responsible for the biosynthesis of pyriculols (Furuyama et al., 2021a; Jacob et al., 2017). It has been suggested that aldehyde-type analogs are biosynthesized first and converted to alcohol-type analogs by a reduction reaction (Tanaka et al., 2011b). The gene (*MGG_10961*/*MoC19OXR1*/*PYC10*) responsible for the oxidation of alcohol-type analogs to aldehyde-type analogs has been reported (Jacob et al., 2017), although the gene catalyzing the reverse reductive reaction has not yet been identified. We recently identified the gene (*MGG_16813*/*PYC7*) for this reverse reaction from aldehyde-type analogs to alcohol-type analogs (Furuyama et al., 2021a) (Fig. 8). Furthermore, previous studies have predicted that alcohol-type analogs are biosynthesized via aldehyde-type analogs. We indicated that aldehyde-type analogs are biosynthesized via alcohol-type analogs (Fig. 8), in contrast to the previous prediction. Similarly, a recent study has shown that a pyriculol-related compound, flavoglaucin (Fig. 7B), is biosynthesized via alcohol intermediates in *Aspergillus ruber* (Nies et al., 2020). We suggest that the rice blast fungus controls the amount of pyriculol analogs using two oxidoreductases, PYC10 and PYC7, thereby controlling the bioactivity of the phytotoxin.

*Neurospora crassa* produces sordaria (Fig. 7B), which shows high structural similarity to pyriculols. A sordarial biosynthetic mechanism has also been proposed (Zhao et al., 2019). The sordarial BGC is highly similar to the pyriculol BGC and has a *PYC10* homolog (*srdI*) and a *PYC7* homolog (*NCU02930*). *N. crassa* may also control the amount of sordarial analogs using two oxidoreductases homologous to PYC10 and PYC7. In addition, it has been proposed that an aldehyde-type intermediate is released from PKS (SrdA) and cyclized by SrdC/D/E. The aldehyde-type intermediate is proposed to be converted by SrbB and SrdG to yield a sordarial. In this pathway, an alcohol-type intermediate is thought to be produced from an aldehyde-type compound by an endogenous reductase, which is expected to be a PYC7 homolog. We assume that an alcohol-type compound is the release product from PKS (SrdA), and an aldehyde-type compound will be produced using the PYC10 homolog SrdI.

The PKS gene (*MGG_10912*/*MoPKS19*/*PYC1*) knockout strain extract failed to induce phytotoxic lesions in rice leaves, suggesting that pyriculols are the sole lesion-inducing compounds produced by the wild-type strain under the culture conditions used (Jacob et al., 2017). Importantly, the PKS gene knockout strain is as pathogenic as the wild-type strain, demonstrating that pyriculols are not needed for infection (Jacob et al., 2017). Dihydropyriculol is thought to be an inactive analog of the phytotoxin, pyriculol. We found that cycloheximide, an antibiotic with protein biosynthesis inhibitory activity, can induce the production of dihydropyriculol (Furuyama et al., 2021b). We revealed that pyriculol and dihydropyriculol have potent growth inhibitory activity against the actinomycete *S. griseus*, which is a cycloheximide producer. Pyriculol inhibits the growth of *P. oryzae*. Localization analysis of dihydropyriculol indicated that this compound can reach *S. griseus* under confrontation culture. These data suggest that dihydropyriculol can be used as a chemical weapon against *S. griseu*s. Pyriculols may be involved in microbe–microbe interactions. Each pyriculol analog may have different roles, and *P. oryzae* may control its biosynthesis to produce a suitable analog, depending on the ecological context.

## Other Secondary Metabolites

Penicillin G production in *P. oryzae* has been reported recently (Saha et al., 2020). Overexpression of a *laeA* homolog (*MoLAEA*/*MGG_07964*) induced the production of penicillin G compared to the parental strain. In contrast, the knockdown strain of this gene did not produce penicillin G. The NRPS gene (*MGG_14767*) was predicted to be involved in penicillin G biosynthesis although it is characteristically different from the *acvA* (*pcbAB*) gene encoding ACV synthetase. *MGG_14767* was upregulated threefold in the *MoLAEA*-overexpression strain, whereas it was downregulated 3.8-fold in the knockdown strain. Transcriptomic analysis showed that MoLAEA regulates the expression of secondary metabolism genes. Interestingly, the *laeA* homolog (*MoLAEA*/*MGG_07964*) is different from the *laeA* homolog (*PoLAE1*/*MGG_01233*) for TeA production control. Another *laeA* homolog (*PoLAE2*/*MGG_08161*) has also been reported to affect the cAMP signaling pathway and appressorium formation (Prajanket et al., 2020). Multiple *laeA* homologs may be involved in controlling secondary metabolite production.

*ACE1* (*MGG_12447*) is a secondary metabolism gene that encodes a PKS–NRPS hybrid enzyme (Böhnert et al., 2004). However, the chemical structure of the final product is unknown. *ACE1* was identified as an avirulence gene, and *the P. oryzae* isolate carrying the *ACE1* gene is specifically recognized by rice cultivars carrying the resistance gene *Pi33*. Defense responses are activated by this recognition in resistant plants. The secondary metabolite whose synthesis is dependent on ACE1 is predicted to be recognized by resistant rice plants. *ACE1* is located in a gene cluster consisting of 15 genes that show a rice-infection-specific expression pattern (Collemare et al., 2008b). Of the 15 genes, 14 genes were predicted to be involved in secondary metabolism because they encode for proteins such as a second PKS–NRPS (SYN2), two enoyl reductases (RAP1 and RAP2), and a putative Zn(II)_2_Cys_6_-type transcription factor (BC2). Heterologous expression of both *ACE1* and *RAP1* in *Aspergillus oryzae* enables the production of an amide compound that is similar to the PKS–NRPS-derived backbone of cytochalasin (Song et al., 2015). However, bioactivity analysis indicated that the produced compound was not responsible for *ACE1*-mediated avirulence. These data suggest that the final active product may be a cytochalasin-like compound.

*ABM* (*MGG_04777*) is a monooxidase gene located in a putative secondary metabolite BGC with a PKS gene (*MGG_04775*) (Patkar et al., 2015). Although the role of Abm (antibiotic biosynthesis monooxygenase) in this gene cluster is unclear, it can oxidize endogenous free jasmonic acid (JA) into 12OH-JA in *P. oryzae*, which is secreted during infection and helps evade the defense response by inhibiting JA-signaling induction. Loss of Abm in *P. oryzae* causes accumulation of methyl JA, which induces host defense and blocks fungal infection. In addition, Abm itself is secreted after infection and is predicted to convert plant JA into 12OH-JA to facilitate host colonization.

*P. oryzae* also produces other plant hormones, such as abscisic acid (ABA) (Jiang et al., 2010), auxins (indole-3-acetic acid [IAA]) (Tanaka et al., 2011a), and cytokinins (CKs) (Jiang et al., 2013), and their biosynthetic genes for ABA and CKs have been described (Chanclud et al., 2016; Spence et al., 2015). Gene disruption of *MoABA4*/*MGG_07514*, a homolog of the *Botrytis cinerea ABA4* gene responsible for ABA biosynthesis, reduces ABA levels by twofold (Spence et al., 2015). The virulence of the Δ*Moaba4* strain was strongly reduced, suggesting that ABA contributes to the virulence of *P. oryzae. CKS1*/*MGG_04857* encodes a putative tRNA-isopentenyl transferase required for CK biosynthesis (Chanclud et al., 2016). The interaction between the Δ*csk1* strain and rice plants was analyzed. This analysis suggested that *P. oryzae*-derived CKs are required for full virulence by affecting nutrient distribution, fungal oxidative stress tolerance, and rice defenses.

## Conclusions

*P. oryzae* has numerous secondary metabolism genes, and some secondary metabolites are expected to be required for rice infection. In this review, we focused on the biosynthesis and biological roles of secondary metabolites in this fungus. Five groups of secondary metabolites (melanin, TeA, nectriapyrones, pyriculols, and penicillin G) have been indicated to be produced by *P. oryzae*. Biosynthetic genes for three (TeA, nectryapyrone, and penicillin G) of the five groups of secondary metabolites were identified by activating secondary metabolism. TeA and nectryapyrones were induced by genetic modification of TCS factors. Penicillin G was induced by overexpression of a *laeA* homolog. Activation of secondary metabolism is a useful method for the identification of secondary metabolites and their biosynthetic genes. DHN-melanin is a well-characterized secondary metabolite that is required for rice infection. Unexpectedly, identification and analysis of the biosynthetic genes showed that three groups of secondary metabolites (TeA, nectriapyrones, and pyriculols) are not required for rice infection. Nectriapyrones, pyriculols, and penicillin G show antibacterial activity and are predicted to be involved in the interactions with bacteria. Control of plant pathogenic fungi is important in agriculture. Elucidating the roles of secondary metabolites in plant pathogenic fungi will help in the development of agrochemicals.

## Data Availability

Experimental data are provided in the manuscript. Authors agree to provide any other data if requested.

## References

[bib1] Abramson H. N. , Wormser H. C. (1981). Synthesis of nectriapyrone. Journal of Heterocyclic Chemistry, 18(2), 363–366. 10.1002/jhet.5570180227

[bib2] Andolfi A. , Boari A., Evidente M., Cimmino A., Vurro M., Ash G., Evidente A. (2015). Gulypyrones A and B and phomentrioloxins B and C produced by *Diaporthe gulyae*, a potential mycoherbicide for saffron thistle (*Carthamus lanatus)*. Journal of Natural Products, 78(4), 623–629. 10.1021/np500570h 25700035

[bib3] Aoki Y. , Matsumoto D., Kawaide H., Natsume M. (2011). Physiological role of germicidins in spore germination and hyphal elongation in Streptomyces coelicolor A3(2). The Journal of Antibiotics, 64(9), 607–611. 10.1038/ja.2011.59 21792209

[bib4] Asam S. , Rychlik M. (2013). Potential health hazards due to the occurrence of the mycotoxin tenuazonic acid in infant food. European Food Research and Technology, 236(3), 491–497. 10.1007/s00217-012-1901-x

[bib5] Avent A. G. , Hanson J. R., Truneh A. (1992). Two pyrones from *Gliocladium vermoesenii*. Phytochemistry, 31(3), 1065–1066. 10.1016/0031-9422(92)80079-T

[bib6] Aver'yanov A. A. , Lapikova V. P., Lebrun M. H. (2007). Tenuazonic acid, toxin of rice blast fungus, induces disease resistance and reactive oxygen production in plants. Russian Journal of Plant Physiology, 54(6), 749–754. 10.1134/S1021443707060052

[bib7] Banba S. , Hamada T., Araki N., Ebihara K. (2017). Synthesis and activities of tolprocarb derivatives against *Pyricularia oryzae*: Relationships among the activities for polyketide synthase, melanin biosynthesis, and rice blast. Journal of Pesticide Science, 42(2), 25–31. 10.1584/jpestics.D16-100 30363093 PMC6140631

[bib8] Bayram O. , Krappmann S., Ni M., Bok J. W., Helmstaedt K., Valerius O., Braus-Stromeyer S., Kwon N. J., Keller N. P., Yu J. H., Braus G. H. (2008). VelB/VeA/LaeA complex coordinates light signal with fungal development and secondary metabolism. Science, 320(5882), 1504–1506. 10.1126/science.1155888 18556559

[bib9] Bell A. A. , Wheeler M. H. (1986). Biosynthesis and functions of fungal melanins. Annual Review of Phytopathology, 24(1), 411–451. 10.1111/nph.16398

[bib10] Bjørk P. K. , Rasmussen S. A., Gjetting S. K., Havshøi N. W., Petersen T. I., Ipsen J., Larsen T. O., Fuglsang A. T. (2020). Tenuazonic acid from Stemphylium loti inhibits the plant plasma membrane H(+) -ATPase by a mechanism involving the C-terminal regulatory domain. New Phytologist, 226(3), 770–784. 10.1128/aem.01979-06 PMC7187312 31880817

[bib11] Blodgett J. A. , Oh D. C., Cao S., Currie C. R., Kolter R., Clardy J. (2010). Common biosynthetic origins for polycyclic tetramate macrolactams from phylogenetically diverse bacteria. Proceedings of the National Academy of Sciences, 107(26), 11692–11697. 10.1042/bj0780412 PMC2900643 20547882

[bib12] Boettger D. , Hertweck C. (2013). Molecular diversity sculpted by fungal PKS-NRPS hybrids. Chembiochem, 14(1), 28–42. 10.1002/cbic.201200624 23225733

[bib13] Böhnert H. U. , Fudal I., Dioh W., Tharreau D., Notteghem J.-L., Lebrun M.-H. (2004). A putative polyketide synthase/peptide synthetase from *Magnaporthe grisea* signals pathogen attack to resistant rice. The Plant Cell, 16(9), 2499–2513. 10.1105/tpc.104.022715 15319478 PMC520948

[bib14] Bok J. W. , Chung D., Balajee S. A., Marr K. A., Andes D., Nielsen K. F., Frisvad J. C., Kirby K. A., Keller N. P. (2006). GliZ, a transcriptional regulator of gliotoxin biosynthesis, contributes to *Aspergillus fumigatus* virulence. Infection and Immunity, 74(12), 6761–6768. 10.1128/iai.00780-06 17030582 PMC1698057

[bib15] Bok J. W. , Keller N. P. (2016). 2 insight into fungal secondary metabolism from ten years of LaeA research. In Hoffmeister D. (Ed.), Biochemistry and molecular biology (pp. 21–29). Springer International Publishing. 10.1007/978-3-319-27790-5_2

[bib16] Brachmann A. O. , Brameyer S., Kresovic D., Hitkova I., Kopp Y., Manske C., Schubert K., Bode H. B., Heermann R. (2013). Pyrones as bacterial signaling molecules. Nature Chemical Biology, 9(9), 573–578. 10.1038/nchembio.1295 23851573

[bib17] Brakhage A. A. (2013). Regulation of fungal secondary metabolism. Nature Reviews Microbiology, 11(1), 21–32. 10.1038/nrmicro2916 23178386

[bib18] Burkhardt I. , Dickschat J. S. (2018). Synthesis and absolute configuration of natural 2-Pyrones. European Journal of Organic Chemistry, 2018(24), 3144–3157. 10.1038/nrmicro2916

[bib19] Butler M. J. , Day A. W. (1998). Fungal melanins: A review. Canadian Journal of Microbiology, 44(12), 1115–1136. 10.1038/nrmicro2916

[bib20] Cai R. , Chen S., Liu Z., Tan C., Huang X., She Z. (2017). A new alpha-pyrone from the mangrove endophytic fungus Phomopsis sp. HNY29-2B. Natural Product Research, 31(2), 124–130. 10.1080/14786419.2016.1214833 27687677

[bib21] Catlett N. L. , Yoder O. C., Turgeon B. G. (2003). Whole-genome analysis of two-component signal transduction genes in fungal pathogens. Eukaryotic Cell, 2(6), 1151–1161. 10.1080/14786419.2016.1214833 14665450 PMC326637

[bib22] Chanclud E. , Kisiala A., Emery N. R., Chalvon V., Ducasse A., Romiti-Michel C., Gravot A., Kroj T., Morel J. B. (2016). Cytokinin production by the rice blast fungus is a pivotal requirement for full virulence. PLOS Pathogens, 12(2), e1005457. 10.1371/journal.ppat.1005457 26900703 PMC4765853

[bib23] Chen S. , Kim C., Lee J. M., Lee H. A., Fei Z., Wang L., Apel K. (2015). Blocking the QB-binding site of photosystem II by tenuazonic acid, a non-host-specific toxin of Alternaria alternata, activates singlet oxygen-mediated and EXECUTER-dependent signalling in Arabidopsis. Plant, Cell & Environment, 38(6), 1069–1080. 10.1111/pce.12462 25292361

[bib24] Chen S. , Qiang S. (2017). Recent advances in tenuazonic acid as a potential herbicide. Pesticide Biochemistry and Physiology, 143, 252–257. 10.1016/j.pestbp.2017.01.003 29183600

[bib25] Chen S. , Xu X., Dai X., Yang C., Qiang S. (2007). Identification of tenuazonic acid as a novel type of natural photosystem II inhibitor binding in Q(B)-site of *Chlamydomonas reinhardtii*. Biochimica et Biophysica Acta (BBA) - Bioenergetics, 1767(4), 306–318. 10.1016/j.bbabio.2007.02.007 17379181

[bib26] Chen S. , Yin C., Qiang S., Zhou F., Dai X. (2010). Chloroplastic oxidative burst induced by tenuazonic acid, a natural photosynthesis inhibitor, triggers cell necrosis in Eupatorium adenophorum Spreng. Biochimica et Biophysica Acta (BBA) - Bioenergetics, 1797(3), 391–405. 10.1016/j.bbabio.2009.12.007 20026008

[bib27] Claydon N. , Grove J. F., Pople M. (1985). Elm bark beetle boring and feeding deterrents from Phomopsis oblonga. Phytochemistry, 24(5), 937–943. 10.1016/S0031-9422(00)83157-X

[bib28] Collemare J. , Billard A., Bohnert H. U., Lebrun M. H. (2008). Biosynthesis of secondary metabolites in the rice blast fungus Magnaporthe grisea: The role of hybrid PKS-NRPS in pathogenicity. Mycological Research, 112(2), 207–215. 10.1016/j.mycres.2007.08.003 18272356

[bib29] Collemare J. , Pianfetti M., Houlle A. E., Morin D., Camborde L., Gagey M. J., Barbisan C., Fudal I., Lebrun M. H., Bohnert H. U. (2008). Magnaporthe grisea avirulence gene ACE1 belongs to an infection-specific gene cluster involved in secondary metabolism. New Phytologist, 179(1), 196–208. 10.1111/j.1469-8137.2008.02459.x 18433432

[bib30] Dean R. A. , Talbot N. J., Ebbole D. J., Farman M. L., Mitchell T. K., Orbach M. J., Thon M., Kulkarni R., Xu J. R., Pan H., Read N. D., Lee Y. H., Carbone I., Brown D., Oh Y. Y., Donofrio N., Jeong J. S., Soanes D. M., Djonovic S., Birren B. W. (2005). The genome sequence of the rice blast fungus Magnaporthe grisea. Nature, 434(7036), 980–986. 10.1111/j.1469-8137.2008.02459.x 15846337

[bib31] de Jong J. C. , McCormack B. J., Smirnoff N., Talbot N. J. (1997). Glycerol generates turgor in rice blast. Nature, 389(6648), 244–245. 10.1038/nature03449

[bib32] Du L. , Sánchez C., Shen B. (2001). Hybrid peptide-polyketide natural products: Biosynthesis and prospects toward engineering novel molecules. Metabolic Engineering, 3(1), 78–95. 10.1006/mben.2000.0171 11162234

[bib33] Dutta S. , Whicher J. R., Hansen D. A., Hale W. A., Chemler J. A., Congdon G. R., Narayan A. R. H., Håkansson K., Sherman D. H., Smith J. L., Skiniotis G. (2014). Structure of a modular polyketide synthase. Nature, 510(7506), 512–517. 10.1038/nature13423 24965652 PMC4278352

[bib34] Eley K. L. , Halo L. M., Song Z., Powles H., Cox R. J., Bailey A. M., Lazarus C. M., Simpson T. J. (2007). Biosynthesis of the 2-pyridone tenellin in the insect pathogenic fungus Beauveria bassiana. Chembiochem, 8(3), 289–297. 10.1002/cbic.200600398 17216664

[bib35] Eliahu N. , Igbaria A., Rose M. S., Horwitz B. A., Lev S. (2007). Melanin biosynthesis in the maize pathogen Cochliobolus heterostrophus depends on two mitogen-activated protein kinases, Chk1 and Mps1, and the transcription factor Cmr1. Eukaryotic Cell, 6(3), 421–429. 10.1128/ec.00264-06 17237364 PMC1828933

[bib36] Evidente A. , Rodeva R., Andolfi A., Stoyanova Z., Perrone C., Motta A. (2011). Phytotoxic polyketides produced by Phomopsis foeniculi, a strain isolated from diseased Bulgarian fennel. European Journal of Plant Pathology, 130(2), 173–182. 10.1007/s10658-011-9743-0

[bib37] Fisch K. M. (2013). Biosynthesis of natural products by microbial iterative hybrid PKS–NRPS. RSC Advances, 3(40), 18228–18247. 10.1039/C3RA42661K

[bib38] Fischbach M. A. , Walsh C. T. (2006). Assembly-line enzymology for polyketide and nonribosomal Peptide antibiotics: Logic, machinery, and mechanisms. Chemical Reviews, 106(8), 3468–3496. 10.1021/cr0503097 16895337

[bib39] Furuyama Y. , Motoyama T., Nogawa T., Hayashi T., Hirota H., Kiyota H., Kamakura T., Osada H. (2021a). Controlling the production of phytotoxin pyriculol in Pyricularia oryzae by aldehyde reductase. Bioscience, Biotechnology, and Biochemistry, 85(1), 126–133. 10.1093/bbb/zbaa035 33577666

[bib40] Furuyama Y. , Motoyama T., Nogawa T., Kamakura T., Osada H. (2021b). Dihydropyriculol produced by Pyricularia oryzae inhibits the growth of Streptomyces griseus. Bioscience, Biotechnology, and Biochemistry.85,(5),1290–1293. 10.1093/bbb/zbab021 33784739

[bib41] Gao X. , Haynes S. W., Ames B. D., Wang P., Vien L. P., Walsh C. T., Tang Y. (2012). Cyclization of fungal nonribosomal peptides by a terminal condensation-like domain. Nature Chemical Biology, 8(10), 823–830. 10.1038/nchembio.1047 22902615 PMC3505271

[bib42] Gerc A. J. , Song L., Challis G. L., Stanley-Wall N. R., Coulthurst S. J. (2012). The insect pathogen serratia marcescens Db10 uses a hybrid non-ribosomal peptide synthetase-polyketide synthase to produce the antibiotic althiomycin. PLoS One, 7(9), e44673. 10.1371/journal.pone.0044673 23028578 PMC3445576

[bib43] Gitterman C. O. (1965). Antitumor, cytotoxic, and antibacterial activities of tenuazonic acid and congeneric tetramic acids. Journal of Medicinal Chemistry, 8(4), 483–486. 10.1021/jm00328a015 4956844

[bib44] Gong T. , Zhen X., Li B.-J., Yang J.-L., Zhu P. (2015). Two new monoterpenoid α-pyrones from a fungus Nectria sp. HLS206 associated with the marine sponge Gelliodes carnosa. Journal of Asian Natural Products Research, 17(6), 633–637. 10.1080/10286020.2015.1040778 26001271

[bib45] Gross M. , Curtui V., Ackermann Y., Latif H., Usleber E. (2011). Enzyme immunoassay for tenuazonic acid in apple and tomato products. Journal of Agricultural and Food Chemistry, 59(23), 12317–12322. 10.1021/jf203540y 22054343

[bib46] Guimaraes D. O. , Borges W. S., Kawano C. Y., Ribeiro P. H., Goldman G. H., Nomizo A., Thiemann O. H., Oliva G., Lopes N. P., Pupo M. T. (2008). Biological activities from extracts of endophytic fungi isolated from Viguiera arenaria and Tithonia diversifolia. FEMS Immunology & Medical Microbiology, 52(1), 134–144. 10.1111/j.1574-695X.2007.00354.x 18081849

[bib47] Hagiwara H. , Ezaki R., Hamada T., Tsuda M., Ebihara K. (2019). Development of a novel fungicide, tolprocarb. Journal of Pesticide Science, 44(3), 208–213. 10.1584/jpestics.J19-01 31530978 PMC6718353

[bib48] Hammerschmidt L. , Debbab A., Ngoc T. D., Wray V., Hemphil C. P., Lin W., Broetz-Oesterhelt H., Kassack M. U., Proksch P., Aly A. H. (2014). Polyketides from the mangrove-derived endophytic fungus Acremonium strictum. Tetrahedron Letters., 55(24), 3463–3468. 10.1016/j.tetlet.2014.04.063

[bib49] Hashimoto M. , Nonaka T., Fujii I. (2014). Fungal type III polyketide synthases. Natural Product Reports, 31(10), 1306–1317. 10.1039/c4np00096j 25182423

[bib50] He W.-J. , Mai Y.-X., Lin X.-P., Liao S.-R., Yang B., Wang J.-F., Liu Y., He W.-J., Zhou X.-J., Qin X.-C., Zhang T., Tu Z.-C., Liu Y. (2016). Quinone/hydroquinone meroterpenoids with antitubercular and cytotoxic activities produced by the sponge-derived fungus Gliomastix sp. ZSDS1-F7. Natural Product Research, 1–6. 10.1039/c4np00096j 27417331

[bib51] Hertweck C. (2009). The biosynthetic logic of polyketide diversity. Angewandte Chemie International Edition, 48(26), 4688–4716. 10.1002/anie.200806121 19514004

[bib52] Howard R. J. , Ferrari M. A. (1989). Role of melanin in appressorium function. Experimental Mycology, 13(4), 403–418. 10.1002/anie.200806121

[bib53] Howard R. J. , Ferrari M. A., Roach D. H., Money N. P. (1991). Penetration of hard substrates by a fungus employing enormous turgor pressures. Proceedings of the National Academy of Sciences, 88(24), 11281–11284. 10.1002/anie.200806121 PMC53118 1837147

[bib54] Howard R. J. , Valent B. (1996). Breaking and entering: Host penetration by the fungal rice blast pathogen Magnaporthe grisea. Annual Review of Microbiology, 50(1), 491–512. 10.1146/annurev.micro.50.1.491 8905089

[bib55] Iwasaki S. , Muro H., Sasaki K., Nozoe S., Okuda S., Sato Z. (1973). Isolations of phytotoxic substances produced by pyricularia oryzae cavara. Tetrahedron Letters, 14(37), 3537–3542. 10.1016/S0040-4039(01)86964-1

[bib56] Jacob S. , Grötsch T., Foster A. J., Schüffler A., Rieger P. H., Sandjo L. P., Liermann J. C., Opatz T., Thines E. (2017). Unravelling the biosynthesis of pyriculol in the rice blast fungus Magnaporthe oryzae. Microbiology (Reading, England), 163(4), 541–553. 10.1099/mic.0.000396 PMC5817273 27902426

[bib57] Janevska S. , Arndt B., Niehaus E. M., Burkhardt I., Rosler S. M., Brock N. L., Humpf H. U., Dickschat J. S., Tudzynski B. (2016). Gibepyrone biosynthesis in the rice pathogen fusarium fujikuroi is facilitated by a small polyketide synthase gene cluster. Journal of Biological Chemistry, 291(53), 27403–27420. 10.1074/jbc.M116.753053 PMC5207165 27856636

[bib58] Jiang C. J. , Shimono M., Sugano S., Kojima M., Liu X., Inoue H., Sakakibara H., Takatsuji H. (2013). Cytokinins act synergistically with salicylic acid to activate defense gene expression in rice. Molecular Plant-Microbe Interactions, 26(3), 287–296. 10.1094/mpmi-06-12-0152-r 23234404

[bib59] Jiang C. J. , Shimono M., Sugano S., Kojima M., Yazawa K., Yoshida R., Inoue H., Hayashi N., Sakakibara H., Takatsuji H. (2010). Abscisic acid interacts antagonistically with salicylic acid signaling pathway in rice-Magnaporthe grisea interaction. Molecular Plant-Microbe Interactions, 23(6), 791–798. 10.1094/mpmi-23-6-0791 20459318

[bib60] Kaku K. , Takagaki M., Shimizu T., Nagayama K. (2003). Diagnosis of dehydratase inhibitors in melanin biosynthesis inhibitor (MBI-D) resistance by primer-introduced restriction enzyme analysis in scytalone dehydratase gene of Magnaporthe grisea. Pest Management Science, 59(8), 843–846. 10.1002/ps.742 12916764

[bib61] Kao C. M. , Pieper R., Cane D. E., Khosla C. (1996). Evidence for two catalytically independent clusters of active sites in a functional modular polyketide synthase. Biochemistry, 35(38), 12363–12368. 10.1021/bi9616312 8823171

[bib62] Keller N. P. , Turner G., Bennett J. W. (2005). Fungal secondary metabolism - From biochemistry to genomics. Nature Reviews Microbiology, 3(12), 937–947. 10.1038/nrmicro1286 16322742

[bib63] Kono Y. , Sekido S., Yamaguchi I., Kondo H., Suzuki Y., Neto G. C., Sakurai A., Yaegashi H. (1991). Structures of two novel pyriculol-related compounds and identification of naturally produced epipyriculol from Pyricularia oryzae. Agricultural and Biological Chemistry, 55, 2785–2791. 10.1080/00021369.1991.10871037

[bib64] Lacriola C. J. , Falk S. P., Weisblum B. (2020). Rice-induced secondary metabolite gene expression in Aspergillus nidulans. Journal of Industrial Microbiology and Biotechnology, 47(12), 1109–1116. 10.1007/s10295-020-02328-x 33210271

[bib65] Lebrun M. H. , Dutfoy F., Gaudemer F., Kunesch G., Gaudemer A. (1990). Detection and quantification of the fungal phytotoxin tenuazonic acid produced by Pyricularia oryzae. Phytochemistry, 29(12), 3777–3783. 10.1016/0031-9422(90)85330-I

[bib66] Lebrun M. H. , Nicolas L., Boutar M., Gaudemer F., Ranomenjanahary S., Gaudemer A. (1988). Relationships between the structure and the phytotoxicity of the fungal toxin tenuazonic acid. Phytochemistry, 27(1), 77–84. 10.1016/0031-9422(88)80594-6

[bib67] Lee I.-K. , Yun B.-S., Oh S., Kim Y.-H., Lee M.-K., Yoo I.-D. (1999). 5-Methylmellein and nectriapyrone, two new monoamine oxidase inhibitors. Medical Science Research, 27, 463–465. 10.1016/0031-9422(88)80594-6

[bib68] Lohrey L. , Marschik S., Cramer B., Humpf H. U. (2013). Large-scale synthesis of isotopically labeled 13C2-tenuazonic acid and development of a rapid HPLC-MS/MS method for the analysis of tenuazonic acid in tomato and pepper products. Journal of Agricultural and Food Chemistry, 61(1), 114–120. 10.1021/jf305138k 23230907

[bib69] Lu X. , Xu N., Dai H.-F., Mei W.-L., Yang Z.-X., Pei Y.-H. (2009). Three new compounds from endophytic fungus L10 of Cephalotaxus hainanensis. Journal of Asian Natural Products Research, 11(5), 397–400. 10.1080/10286020902819947 19504381

[bib70] Lundqvist T. , Rice J., Hodge C. N., Basarab G. S., Pierce J., Lindqvist Y. (1994). Crystal structure of scytalone dehydratase–a disease determinant of the rice pathogen, Magnaporthe grisea. Structure (London, England), 2(10), 937–944. 10.1080/10286020902819947 7866745

[bib71] Macheleidt J. , Mattern D. J., Fischer J., Netzker T., Weber J., Schroeckh V., Valiante V., Brakhage A. A. (2016). Regulation and role of fungal secondary metabolites. Annual Review of Genetics, 50(1), 371–392. 10.1146/annurev-genet-120215-035203 27732794

[bib72] Maeda K. , Izawa M., Nakajima Y., Jin Q., Hirose T., Nakamura T., Koshino H., Kanamaru K., Ohsato S., Kamakura T., Kobayashi T., Yoshida M., Kimura M. (2017). Increased metabolite production by deletion of an HDA1-type histone deacetylase in the phytopathogenic fungi, *Magnaporthe oryzae* (*Pyricularia oryzae*) and *Fusarium asiaticum*. Letters in Applied Microbiology, 65(5), 446–452. 10.1111/lam.12797 28862744

[bib73] McGlacken G. P. , Fairlamb I. J. (2005). 2-Pyrone natural products and mimetics: Isolation, characterisation and biological activity. Natural Product Reports, 22(3), 369–385. 10.1039/b416651p 16010346

[bib74] Meister J. , Weber D., Martino V., Sterner O., Anke T. (2007). Phomopsidone, a novel depsidone from an endophyte of the medicinal plant Eupatorium arnottianum. Zeitschrift für Naturforschung C, 62(1-2), 11–15. 10.1515/znc-2007-1-202 17425098

[bib75] Miller F. A. , Rightsel W. A., Sloan B. J., Ehrlich J., French J. C., Bartz Q. R. (1963). Antiviral activity of tenuazonic acid. Nature, 200(4913), 1338–1339. 10.1038/2001338a0 14098497

[bib76] Money N. P. , Howard R. J. (1996). Confirmation of a link between fungal pigmentation, turgor pressure, and pathogenicity using a new method of turgor measurement. Fungal Genetics & Biology, 20, 217–227. 10.1038/2001338a0

[bib77] Motoyama T. , Nogawa T., Hayashi T., Hirota H., Osada H. (2019). Induction of nectriapyrone biosynthesis in the rice blast fungus pyricularia oryzae by disturbance of the two-component signal transduction system. Chembiochem, 20(5), 693–700. 10.1002/cbic.201800620 30443971

[bib78] Motoyama T. , Ochiai N., Morita M., Iida Y., Usami R., Kudo T. (2008). Involvement of putative response regulator genes of the rice blast fungus Magnaporthe oryzae in osmotic stress response, fungicide action, and pathogenicity. Current Genetics, 54(4), 185–195. 10.1007/s00294-008-0211-0 18726099

[bib79] Motoyama T. , Osada H. (2016). Biosynthetic approaches to creating bioactive fungal metabolites: Pathway engineering and activation of secondary metabolism. Bioorganic & Medicinal Chemistry Letters, 26(24), 5843–5850. 10.1016/j.bmcl.2016.11.013 27865702

[bib80] Motoyama T. , Yamaguchi I. (2003). Fungicides, melanin biosynthesis inhibitors. In: Plimmer J. R., Gammon D. W., Ragsdale N. N. (Eds.), Encyclopedia of agrochemicals (Vol. 2, pp. 584–592). Wiley. 10.1002/047126363X.agr102

[bib81] Müller S. , Garcia-Gonzalez E., Mainz A., Hertlein G., Heid N. C., Mösker E., van den Elst H., Overkleeft H. S., Genersch E., Süssmuth R. D. (2014). Paenilamicin: Structure and biosynthesis of a hybrid nonribosomal peptide/polyketide antibiotic from the bee pathogen paenibacillus larvae. Angewandte Chemie International Edition, 53(40), 10821–10825. 10.1002/anie.201404572 25080172

[bib82] Nair M. S. R. , Carey S. T. (1975). Metabolites of pyrenomycetes. II. Nectriapyrone, an antibiotic monoterpenoid. Tetrahedron Letters, 1655–1658. 10.1016/0040-4039(75)85038-6 10.1016/0040-4039(75)85038-6

[bib83] Netzker T. , Fischer J., Weber J., Mattern D. J., Konig C. C., Valiante V., Schroeckh V., Brakhage A. A. (2015). Microbial communication leading to the activation of silent fungal secondary metabolite gene clusters. Frontiers in Microbiology, 6, 299. 10.3389/fmicb.2015.00299 25941517 PMC4403501

[bib84] Nies J. , Ran H., Wohlgemuth V., Yin W. B., Li S. M. (2020). Biosynthesis of the prenylated salicylaldehyde flavoglaucin requires temporary reduction to salicyl alcohol for decoration before reoxidation to the final product. Organic Letters, 22(6), 2256–2260. 10.1021/acs.orglett.0c00440 32134669

[bib85] Ninomiya A. , Urayama S. I., Suo R., Itoi S., Fuji S. I., Moriyama H., Hagiwara D. (2020). Mycovirus-induced tenuazonic acid production in a rice blast fungus *Magnaporthe oryzae*. Frontiers in Microbiology, 11, 1641. 10.3389/fmicb.2020.01641 32765467 PMC7379127

[bib86] Nukina M. , Sassa T., Ikeda M., Umezawa T., Tasaki H. (1981). Pyriculariol, a new phytotoxic metabolite of pyricularia oryzae cavara. Agricultural and Biological Chemistry, 45, 2161–2162. 10.1080/00021369.1981.10864855

[bib87] Ochi K. , Hosaka T. (2013). New strategies for drug discovery: Activation of silent or weakly expressed microbial gene clusters. Applied Microbiology and Biotechnology, 97(1), 87–98. 10.1007/s00253-012-4551-9 23143535 PMC3536979

[bib88] Ostry V. (2008). Alternaria mycotoxins: An overview of chemical characterization, producers, toxicity, analysis and occurrence in foodstuffs. World Mycotoxin Journal, 1(2), 175–188. 10.3920/WMJ2008.x013

[bib89] Patkar R. N. , Benke P. I., Qu Z., Chen Y. Y., Yang F., Swarup S., Naqvi N. I. (2015). A fungal monooxygenase-derived jasmonate attenuates host innate immunity. Nature Chemical Biology, 11(9), 733–740. 10.1038/nchembio.1885 26258762

[bib90] Perrin R. M. , Fedorova N. D., Bok J. W., Cramer R. A. Jr, Wortman J. R., Kim H. S., Nierman W. C., Keller N. P. (2007). Transcriptional regulation of chemical diversity in Aspergillus fumigatus by LaeA. PLOS Pathogens, 3(4), e50. 10.1371/journal.ppat.0030050 17432932 PMC1851976

[bib91] Petersen F. , Zahner H., Metzger J. W., Freund S., Hummel R. P. (1993). Germicidin, an autoregulative germination inhibitor of Streptomyces viridochromogenes NRRL B-1551. The Journal of Antibiotics, 46(7), 1126–1138. 10.1371/journal.ppat.0030050 8360109

[bib92] Prajanket P. , Vu K. T., Arai J., Sornkom W., Abe A., Sone T. (2020). Function of *PoLAE2*, a *laeA* homolog, in appressorium formation and cAMP signal transduction in *Pyricularia oryzae*. Bioscience, Biotechnology, and Biochemistry, 84(11), 2401–2404. 10.1080/09168451.2020.1801379 32729391

[bib93] Proctor R. H. , Hohn T. M., McCormick S. P., Desjardins A. E. (1995). Tri6 encodes an unusual zinc finger protein involved in regulation of trichothecene biosynthesis in Fusarium sporotrichioides. Applied and Environmental Microbiology, 61(5), 1923–1930. 10.1128/aem.61.5.1923-1930.1995 7646028 PMC167455

[bib94] Rosett T. , Sankhala R. H., Stickings C. E., Taylor M. E. U., Thomas R. (1957). Studies in the biochemistry of micro-organisms. 103. Metabolites of *Alternaria tenuis* auct.: Culture filtrate products. Biochemical Journal, 67(3), 390–400. 10.1042/bj0670390 PMC1200169 13479395

[bib95] Rutledge P. J. , Challis G. L. (2015). Discovery of microbial natural products by activation of silent biosynthetic gene clusters. Nature Reviews Microbiology, 13(8), 509–523. 10.1038/nrmicro3496 26119570

[bib96] Saha P. , Ghosh S., Roy-Barman S. (2020). *MoLAEA* regulates secondary metabolism in *Magnaporthe oryzae*. mSphere, 5(2), e00936–19. 10.1128/mSphere.00936-19 32238572 PMC7113587

[bib97] Sawada H. , Sugihara M., Takagaki M., Nagayama K. (2004). Monitoring and characterization of Magnaporthe grisea isolates with decreased sensitivity to scytalone dehydratase inhibitors. Pest Management Science, 60(8), 777–785. 10.1002/ps.858 15307669

[bib98] Schaberle T. F. (2016). Biosynthesis of alpha-pyrones. Beilstein Journal of Organic Chemistry, 12, 571–588. 10.3762/bjoc.12.56 27340449 PMC4901931

[bib99] Shigeura H. T. , Gordon C. N. (1963). The biological activity of tenuazonic acid. Biochemistry, 2(5), 1132–1137. 10.1021/bi00905a039 14087373

[bib100] Shimizu T. , Kinoshita H., Nihira T. (2007). Identification and in vivo functional analysis by gene disruption of ctnA, an activator gene involved in citrinin biosynthesis in Monascus purpureus. Applied and Environmental Microbiology, 73(16), 5097–5103. 10.1128/aem.01979-06 17586673 PMC1950990

[bib101] Siegel D. , Rasenko T., Koch M., Nehls I. (2009). Determination of the Alternaria mycotoxin tenuazonic acid in cereals by high-performance liquid chromatography-electrospray ionization ion-trap multistage mass spectrometry after derivatization with 2,4-dinitrophenylhydrazine. Journal of Chromatography A, 1216(21), 4582–4588. 10.1016/j.chroma.2009.03.063 19361805

[bib102] Silakowski B. , Schairer H. U., Ehret H., Kunze B., Weinig S., Nordsiek G., Brandt P., Blöcker H., Höfle G., Beyer S., Müller R. (1999). New lessons for combinatorial biosynthesis from myxobacteria. The myxothiazol biosynthetic gene cluster of Stigmatella aurantiaca DW4/3-1. Journal of Biological Chemistry, 274(52), 37391–37399. 10.1074/jbc.274.52.37391 10601310

[bib103] Simunovic V. , Zapp J., Rachid S., Krug D., Meiser P., Müller R. (2006). Myxovirescin A biosynthesis is directed by hybrid polyketide synthases/nonribosomal peptide synthetase, 3-hydroxy-3-methylglutaryl-CoA synthases, and trans-acting acyltransferases. Chembiochem, 7(8), 1206–1220. 10.1002/cbic.200600075 16835859

[bib104] Smith E. R. , Fredrickson T. N., Hadidian Z. (1968). Toxic effects of the sodium and the N,N'-dibenzylethylenediamine salts of tenuazonic acid. Cancer Chemotherapy Reports Part 1, 52, 579–585. 10.1002/cbic.200600075 4988460

[bib105] Song Z. , Bakeer W., Marshall J. W., Yakasai A. A., Khalid R. M., Collemare J., Skellam E., Tharreau D., Lebrun M. H., Lazarus C. M., Bailey A. M., Simpson T. J., Cox R. J. (2015). Heterologous expression of the avirulence gene ACE1 from the fungal rice pathogen Magnaporthe oryzae. Chemical Science, 6(8), 4837–4845. 10.1021/ja065247w 29142718 PMC5667575

[bib106] Song L. , Barona-Gomez F., Corre C., Xiang L., Udwary D. W., Austin M. B., Noel J. P., Moore B. S., Challis G. L. (2006). Type III polyketide synthase beta-ketoacyl-ACP starter unit and ethylmalonyl-CoA extender unit selectivity discovered by Streptomyces coelicolor genome mining. Journal of the American Chemical Society, 128(46), 14754–14755. 10.1039/c4sc03707c 17105255 PMC2859292

[bib107] Song Z. , Cox R. J., Lazarus C. M., Simpson T. T. (2004). Fusarin C biosynthesis in Fusarium moniliforme and Fusarium venenatum. Chembiochem, 5(9), 1196–1203. 10.1002/cbic.200400138 15368570

[bib108] Spence C. A. , Lakshmanan V., Donofrio N., Bais H. P. (2015). Crucial roles of abscisic acid biogenesis in virulence of rice blast fungus magnaporthe oryzae. Frontiers in Plant Science, 6, 1082. 10.3389/fpls.2015.01082 26648962 PMC4664623

[bib109] Steyn P. S. , Rabie C. J. (1976). Characterization of magnesium and calcium tenuazonate from Phoma sorghina. Phytochemistry, 15(12), 1977–1979. 10.1016/S0031-9422(00)88860-3

[bib110] Stickings C. E. , Townsend R. J. (1961). Studies in the biochemistry of micro-organisms. 108. Metabolites of Alternaria tenuis Auct.: The biosynthesis of tenuazonic acid. Biochemical Journal, 78(2), 412–418. 10.1042/bj0780412 PMC1205283 16748879

[bib111] Tanaka E. , Koga H., Mori M., Mori M. (2011). Auxin production by the rice blast fungus and its localization in host tissue. Journal of Phytopathology, 159(7-8), 522–530. 10.1111/j.1439-0434.2011.01799.x

[bib112] Tanaka K. , Sasaki A., Cao H.-Q., Yamada T., Igarashi M., Komine I., Nakahigashi H., Minami N., Kuwahara S., Nukina M., Kiyota H. (2011). Synthesis and biotransformation of plausible biosynthetic intermediates of salicylaldehyde-type phytotoxins of rice blast fungus, magnaporthe grisea. European Journal of Organic Chemistry, 2011(31), 6276–6280. 10.1002/ejoc.201100771

[bib113] Tang G. L. , Cheng Y. Q., Shen B. (2004). Leinamycin biosynthesis revealing unprecedented architectural complexity for a hybrid polyketide synthase and nonribosomal peptide synthetase. Chemistry & Biology, 11(1), 33–45. 10.1016/j.chembiol.2003.12.014 15112993

[bib114] Tanovic A. , Samel S. A., Essen L. O., Marahiel M. A. (2008). Crystal structure of the termination module of a nonribosomal peptide synthetase. Science, 321(5889), 659–663. 10.1126/science.1159850 18583577

[bib115] Thines E. , Anke H., Sterner O. (1998). Scytalols A, B, C, and D and other modulators of melanin biosynthesis from Scytalidium sp. 36–93. The Journal of Antibiotics, 51(4), 387–393. 10.7164/antibiotics.51.387 9630860

[bib116] Thompson J. E. , Fahnestock S., Farrall L., Liao D. I., Valent B., Jordan D. B. (2000). The second naphthol reductase of fungal melanin biosynthesis in Magnaporthe grisea: Tetrahydroxynaphthalene reductase. Journal of Biological Chemistry, 275(45), 34867–34872. 10.1074/jbc.M006659200 10956664

[bib117] Trauger J. W. , Kohli R. M., Mootz H. D., Marahiel M. A., Walsh C. T. (2000). Peptide cyclization catalysed by the thioesterase domain of tyrocidine synthetase. Nature, 407(6801), 215–218. 10.1038/35025116 11001063

[bib118] Turkkan M. , Andolfi A., Zonno M. C., Erper I., Perrone C., Cimmino A., Vurro M., Evidente A. (2011). Phytotoxins produced by Pestalotiopsis guepinii, the causal agent of hazelnut twig blight. Phytopathologia Mediterranea., 50, 154–158. 10.1038/35025116

[bib119] Umetsu N. , Kaji J., Tamari K. (1972). Investigation on the toxin production by several blast fungus strains and isolation of tenuazonic acid as a novel toxin. Agricultural and Biological Chemistry, 36(5), 859–866. 10.1080/00021369.1972.10860315

[bib120] Vagstad A. L. , Hill E. A., Labonte J. W., Townsend C. A. (2012). Characterization of a fungal thioesterase having Claisen cyclase and deacetylase activities in melanin biosynthesis. Chemistry & Biology, 19(12), 1525–1534. 10.1016/j.chembiol.2012.10.002 23261597 PMC3530136

[bib121] Vidal-Cros A. , Viviani F., Labesse G., Boccara M., Gaudry M. (1994). Polyhydroxynaphthalene reductase involved in melanin biosynthesis in Magnaporthe grisea. Purification, cDNA cloning and sequencing. European Journal of Biochemistry, 219(3), 985–992. 10.1016/j.chembiol.2012.10.002 8112349

[bib122] Weber D. , Gorzalczany S., Martino V., Acevedo C., Sterner O., Anke T. (2005). Metabolites from endophytes of the medicinal plant Erythrina crista-galli. Zeitschrift für Naturforschung C, 60(5–6), 467–477. 10.1515/znc-2005-5-616 16042349

[bib123] Weissman K. J. (2015). Uncovering the structures of modular polyketide synthases. Natural Product Reports, 32(3), 436–453. 10.1039/C4NP00098F 25310997

[bib124] West A. H. , Stock A. M. (2001). Histidine kinases and response regulator proteins in two-component signaling systems. Trends in Biochemical Sciences, 26(6), 369–376. 10.1039/C4NP00098F 11406410

[bib125] Xi J. , Yang Z., Xu J., Ge M., Chen D. (2012). Study on the metabolites of endophytic fungus Colletotrichum sp. from Elaeagnus umbellata Thunb. Xibei Yaoxue Zazhi, 27, 523–525. 10.3969/j.issn.1004-2407.2012.06.007

[bib126] Xu Z. , Ding L., Hertweck C. (2011). A branched extender unit shared between two orthogonal polyketide pathways in an endophyte. Angewandte Chemie International Edition, 50(20), 4667–4670. 10.1002/anie.201008265 21506215

[bib127] Yamada N. , Motoyama T., Nakasako M., Kagabu S., Kudo T., Yamaguchi I. (2004). Enzymatic characterization of scytalone dehydratase Val75Met variant found in melanin biosynthesis dehydratase inhibitor (MBI-D) resistant strains of the rice blast fungus. Bioscience, Biotechnology, and Biochemistry, 68(3), 615–621. 10.1271/bbb.68.615 15056895

[bib128] Yang Y. H. , Yang D. S., Lei H. M., Li C. Y., Li G. H., Zhao P. J. (2019). Griseaketides A-D, new aromatic polyketides from the pathogenic fungus magnaporthe grisea. Molecules (Basel, Switzerland), 25(1), 72., 10.3390/molecules25010072 PMC6982942 31878244

[bib129] Yin W. , Keller N. P. (2011). Transcriptional regulatory elements in fungal secondary metabolism. Journal of Microbiology (Seoul, Korea), 49, 329–339. 10.1007/s12275-011-1009-1 PMC3714018 21717315

[bib130] Yu J. H. , Butchko R. A., Fernandes M., Keller N. P., Leonard T. J., Adams T. H. (1996). Conservation of structure and function of the aflatoxin regulatory gene aflR from Aspergillus nidulans and A. flavus. Current Genetics, 29(6), 549–555. 10.1128/AAC.00931-06 8662194

[bib131] Yu F. , Zaleta-Rivera K., Zhu X., Huffman J., Millet J. C., Harris S. D., Yuen G., Li X. -C., Du L. (2007). Structure and biosynthesis of heat-stable antifungal factor (HSAF), a broad-spectrum antimycotic with a novel mode of action. Antimicrobial Agents and Chemotherapy, 51(1), 64–72. 10.1007/bf02426959. 17074795 PMC1797680

[bib132] Yun C. S. , Motoyama T., Osada H. (2015). Biosynthesis of the mycotoxin tenuazonic acid by a fungal NRPS-PKS hybrid enzyme. Nature Communications, 6(1), 8758. 10.1038/ncomms9758 PMC4640141 26503170

[bib133] Yun C. S. , Motoyama T., Osada H. (2017). Regulatory mechanism of mycotoxin tenuazonic acid production in pyricularia oryzae. ACS Chemical Biology., 12(9), 2270–2274. 10.1021/acschembio.7b00353 28820236

[bib134] Yun C. S. , Nishimoto K., Motoyama T., Shimizu T., Hino T., Dohmae N., Nagano S., Osada H. (2020). Unique features of the ketosynthase domain in a non-ribosomal peptide synthetase-polyketide synthase hybrid enzyme, tenuazonic acid synthetase 1. Journal of Biological Chemistry. 295(33), 11602–11612. 10.1074/jbc.RA120.013105 PMC7450107 32565425

[bib135] Zhao Z. , Ying Y., Hung Y. S., Tang Y. (2019). Genome mining reveals neurospora crassa can produce the salicylaldehyde sordarial. Journal of Natural Products, 82(4), 1029–1033. 10.1021/acs.jnatprod.8b00983 30908040 PMC6933945

[bib136] Zhu H. , Hua X.-x., Gong T., Pang J., Hou Q., Zhu P. (2013). Hypocreaterpenes A and B, cadinane-type sesquiterpenes from a marine-derived fungus, Hypocreales sp. Phytochemistry Letters, 6(3), 392–396. 10.1016/j.phytol.2013.04.008

